# Bat-Inspired Longevity: Immune Damage Management and Nutritional Modulation for Healthy Aging

**DOI:** 10.3390/ijms27104467

**Published:** 2026-05-16

**Authors:** Sunmin Park, James W. Daily

**Affiliations:** 1 Department of Food and Nutrition, Obesity/Diabetes Research Center, Hoseo University, Asan 31499, Republic of Korea; 2 Department of Bio-Convergence System, Hoseo University, Asan 31499, Republic of Korea; 3 Department of R&D, Daily Manufacturing, Inc., Rockwell, NC 28138, USA; jdaily3@yahoo.com

**Keywords:** bat longevity, inflammaging, autophagy–mitophagy, inflammasome restraint, gut microbiota, nutritional geroscience

## Abstract

The exceptional longevity of bats challenges classical theories of inflammaging and suggests an alternative that improved resilience in responding to pathogens and cellular damage can increase longevity. Accordingly, we have developed the Core Longevity State Vector (CLSV-6) to characterize an expanded explanation for inflammaging that can be predictive of successful aging and used to develop potential strategies for successful aging. Despite high metabolic rates and persistent viral exposure, many bat species have much longer lifespans than would be predicted for mammals of their size. The increased longevity of many bat species is achieved through damage tolerance, regulated inflammasome activity, constitutive basal antiviral defenses, enhanced autophagy–mitophagy, and efficient resolution of inflammation, rather than through heightened inflammatory immunity. The CLSV-6 is introduced as a multidimensional immunotype framework integrating six conserved mechanisms that link bat immunity to bat longevity and to human healthy aging: (1) damage tolerance, (2) autophagy–mitophagy, (3) proteostasis (management of degraded proteins), (4) basal immune readiness without activation, (5) inflammasome regulation, and (6) inflammatory resolution capacity. Together, these mechanisms enable a robust antiviral defense when needed without chronic inflammation. Notably, centenarians converge toward this bat-like configuration. Studies suggest that centenarians often preserve more functional NK cells, better macrophage regulation, and improved anti-inflammatory control, with both bats and humans exhibiting reduced activation of the NLRP3 inflammasome, resulting in greater immune resilience. Building on this framework, functional foods—including polyphenols, fermented foods, and herbal extracts—are proposed as practical strategies to shift human immunity toward bat-like, CLSV-6 immunotype by enhancing cellular quality control, regulating inflammasome activity, strengthening basal antiviral readiness, and supporting inflammatory resolution, thereby redirecting longevity strategies from immune stimulation toward damage containment and repair. This review reframes longevity as an emergent property of integrated immune damage management and provides a mechanistic roadmap for nutritional interventions to engineer healthier human aging inspired by bat immunity.

## 1. Introduction

Aging is a consistent progression of degenerative processes [1] linked to chronic inflammation, known as inflammaging. The accepted “hallmarks of aging are genomic instability, telomere attrition, epigenetic alterations, loss of proteostasis, disabled macroautophagy, deregulated nutrient-sensing, mitochondrial dysfunction, cellular senescence, stem cell exhaustion, altered intercellular communication, chronic inflammation, and dysbiosis. All of these hallmarks share immune dysregulation as a common causative link [1], and in turn, many are major causes of inflammation. Inflammaging is defined as a persistent age-related low-grade inflammation in the absence of overt infection, or inflammaging [2], and is associated with cardiovascular disease, metabolic dysfunction, neurodegeneration, frailty, and reduced survival [3,4,5], yet presents a paradox: centenarians often exhibit low circulating inflammatory markers while maintaining immune competence and infection resistance [6]. It suggests that healthy longevity depends on immune regulation rather than immune intensity [6,7]. Single biomarkers such as interleukin (IL)-6 and C-reactive protein (CRP) are valuable predictors of disease and mortality, but are inconsistent across age groups and fail to distinguish protective immune readiness from maladaptive inflammatory persistence [8,9,10]. Age-associated inflammation can often be better understood as impaired damage handling: when clearance, containment, or resolution becomes inefficient, immune activation persists as a consequence of damage accumulation rather than as an independent driver of aging [11].

Exceptional longevity across long-lived mammals is achieved by preserving immune defense while restraining inflammation, demonstrating that immunity can be physiologically uncoupled from chronic inflammatory pathology [12]. Bats exemplify this principle. Certain species exceed 40 years despite body masses comparable to rodents that survive only 2–3 years, a disparity attributable to adaptations enabling flight, echolocation, and hibernation [13]. Powered flight generates extreme reactive oxygen species (ROS) and cytosolic DNA loads that would cause lethal systemic inflammation in most mammals; bats survived by evolving a “tolerate and clear” strategy—constitutive interferon (IFN)-α, establishing basal antiviral defense without sustained inflammation, and dampened NOD-like receptor pyrin domain-containing protein 3 (*NLRP3*) inflammasome responses limiting IL-1β without compromising viral control [11,14], enhanced autophagy, mitophagy, and DNA repair for upstream damage management [14,15]. The gut microbiomes of many bat species are enriched in *Proteobacteria* rather than the *Firmicutes*/*Bacteroidetes*-dominated communities typical of terrestrial mammals [13,16]. In humans, persistent Proteobacteria expansion is commonly associated with barrier dysfunction and inflammatory disease; however, bats maintain immune tolerance and physiological stability despite this microbiome structure, likely through adaptations involving rapid gut transit, reinforced intestinal integrity, and restrained inflammatory signaling [17]. Long-lived humans appear to converge toward comparable physiological strategies characterized by preserved barrier function, controlled inflammatory execution, and enhanced resilience to chronic stressors [6]. In this review, we identify similarities between the physiological and immunological characteristics of bats and humans. In this review, we identify parallels between the physiological and immunological characteristics of bats and long-lived humans. In some cases, the underlying molecular pathways are directly shared across species, whereas in others, distinct mechanisms converge on similar functional outcomes. By integrating findings from bat immunophysiology and human longevity research, we propose the Core Longevity State Vector (CLSV-6) as a multidimensional immunotype framework that reframes nutritional and biological interventions as modulators of damage handling, immune regulation, and inflammatory resolution.

## 2. Bat Immune System: Unique Features Enabling Inflammation-Resistant Survival

Bats commonly exhibit a distinctive immune architecture that preserves pathogen control while actively restraining inflammatory pathology [18]. Rather than relying on robust immune activation, bat immunity is characterized by selective tuning of innate sensing, constitutive but balanced interferon signaling, dampened inflammasome execution, and superior management of cellular damage [19]. In the animal kingdom, longevity is typically correlated with body mass and metabolic rate, with smaller mammals generally having shorter lifespans. Bats represent a significant evolutionary outlier: while rodents of comparable size typically survive only 2–3 years, certain bat species exceed 40 years—an order-of-magnitude difference that defies standard metabolic scaling laws [20,21] (Table 1).

**Figure 1:**
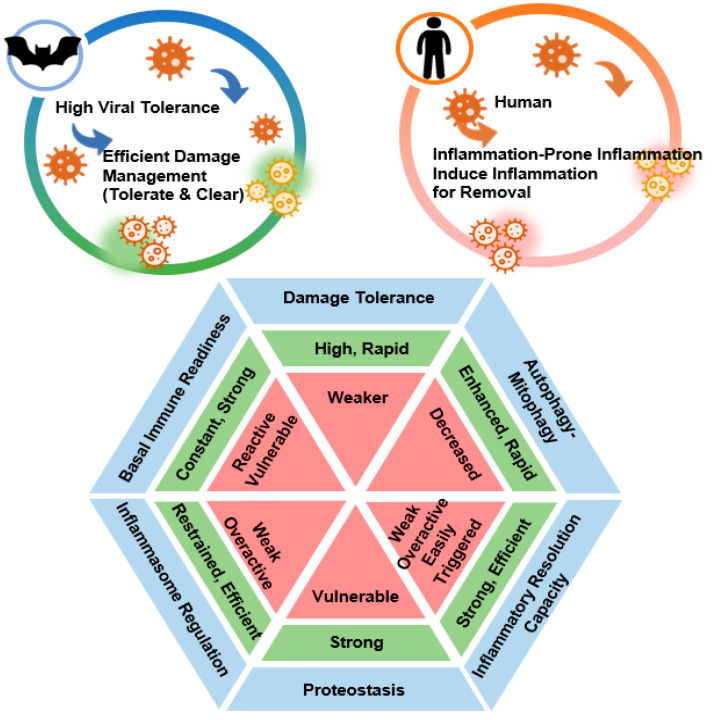
Evolutionary comparison of immune damage management via Core Longevity State Vector (CLSV)-6. This schematic illustrates the evolutionary divergence in damage management between species. The upper panel contrasts the “Tolerate & Clear” strategy of bats, which promotes healthy longevity, against the “Inflammation-Prone” strategy of humans, which results in tissue damage and accelerated aging. The central hexagon (CLSV-6) details the six integrated domains of the CLSV: (1) Damage Tolerance, (2) Autophagy–Mitophagy, (3) Proteostasis, (4) Inflammatory Resolution Capacity, (5) Inflammasome Restraint, and (6) Basal Immune Readiness. Green segments denote the optimized, pro-longevity states characteristic of bats, while red segments denote the reactive, pro-aging states typical of humans. The blue outer ring represents the six regulatory pillars of the Core Longevity State Vector (CLSV)-6. Together, these domains determine the systemic threshold for inflammation and long-term tissue integrity.

This exceptional longevity is intrinsically linked to immune architecture and telomere maintenance. Age-related telomere shortening—a hallmark of aging—is largely absent in the longest-lived bat species, likely reflecting their suppressed inflammatory responses, since inflammation is a major contributor to telomere degradation [22,23]. While other small mammals succumb to oxidative stress and rapid inflammatory aging, bats have naturally evolved with a CLSV-6 immunotype framework to uncouple high metabolic activity from chronic inflammation (Figure 1). Central to this regulation are molecular brakes—including the S358 stimulator of interferon genes (STING) mutation and NLRP3 dampening—that prevent overreaction to self-DNA released during flight, decoupling damage-associated molecular pattern (DAMP) sensing from systemic inflammatory execution, thereby maintaining immune vigilance without chronic inflammatory cost [24].

### 2.1. Innate Immunity in Bats: Modified Sensing Without Overreaction

Innate immune recognition typical in bats shows functional divergence in pattern recognition receptors (PRRs) pathways. Although canonical sensors such as TLRs and cytosolic DNA sensors are conserved across mammals, their downstream inflammatory outputs are restrained in bats [25]. Viral-sensing toll-like receptors (TLRs; TLR3, TLR7, TLR8, TLR9) carry signatures of positive selection, while TLR2-mediated inflammatory signaling is dampened through bat-specific amino acid substitutions that reduce cytokine production without compromising pathogen detection [26].

DNA sensing illustrates the decoupling of detection from inflammation. The S358 STING mutation markedly reduces interferon regulatory factor (IRF3) activation and IFN-β induction following cytosolic DNA sensing [27], confirmed by wild-type rescue experiments [26] and especially relevant given the high oxidative and DNA damage burden of flight. Loss of the Pyrin and HIN domain (PYHIN) sensor family—including absent in melanoma 2 (AIM2) and interferon gamma inducible protein 16 (IFI16)—further eliminates DNA-triggered inflammasome activation [28]. Many bat species exhibit constitutive expression of IFN-stimulated genes, shaped by contraction of the IFNα locus and expansion of *IFNω* genes, which maintains antiviral readiness without episodic hyperactivation [29]. Downstream signaling remains fully functional, as bat cells respond normally to exogenous interferon stimulation, confirming that bat innate immunity prioritizes early containment while minimizing inflammatory escalation [11]. Importantly, the S358 STING attenuation reflects permanent germline evolution—a fixed, high-magnitude genomic change. Dietary modulation of STING signaling operates through considerably weaker and more transient mechanisms.

### 2.2. Inflammasome Dampening and Limited Inflammatory Execution

A hallmark of bat immunity is broad attenuation of inflammasome activation in many species [30]. *NLRP3* responsiveness is often reduced through structural variants and diminished priming, resulting in markedly lower IL-1β secretion despite effective pathogen control [31]; many Old World fruit bats lack *NLRP1* entirely, removing an additional inflammasome pathway [31]. Bats uniquely express activating signal cointegrator-2 (ASC2), a dominant-negative inhibitor of inflammasome assembly that suppresses caspase-1 activation and IL-1β/IL-18 maturation [6]; introduction of bat ASC2 into mouse models significantly reduced inflammatory pathology, directly demonstrating this bat-specific mechanism [32]. Tumor necrosis factor (TNF)-α signaling is constrained—potentially through mutations in the c-Rel binding motif of the TNF promoter—while IL-10 is enhanced, promoting rapid resolution [25]. Inflammation in bats is thus selectively deployed and rapidly terminated rather than chronically amplified.

### 2.3. Autophagy, DNA Repair, and Damage Tolerance

Beyond immune signaling, bats exhibit exceptional upstream damage control. Basal autophagy and mitophagy are elevated, facilitating efficient clearance of damaged organelles, protein aggregates, and intracellular pathogens [33,34]. Comparative genomics reveals positive selection in multiple autophagy and DNA repair genes, supporting enhanced mitochondrial quality control and genomic maintenance [35]. By limiting cytosolic release of mtDNA and oxidized DNA, these systems prevent activation of cyclic GMP-AMP synthase (cGAS)-STING and inflammasome pathways [26,34,36]. Enhanced proteostasis via heat shock protein expression further reduces DAMP accumulation [37]. Collectively, these mechanisms reduce the inflammatory burden by addressing damage at its source rather than responding to it reactively [22].

### 2.4. Gut Microbiome Architecture and Immune–Microbial Tolerance

A defining feature of bat immunity is remarkable tolerance of gut microbial communities that would trigger chronic inflammation in other mammals. Bat intestinal communities of many bat species are dominated by *Proteobacteria* (40–70% of total bacteria) across frugivorous, insectivorous, nectarivorous, and sanguivorous species [38,39]—a composition reflecting flight-related physiological constraints such as rapid gut transit and high metabolic rate, rather than dietary macronutrient content alone. In humans, Proteobacteria dominance is a diagnostic marker of dysbiosis consistently associated with inflammatory bowel disease, metabolic endotoxemia, and systemic inflammation [40]. As Gram-negative bacteria, Proteobacteria produce lipopolysaccharide (LPS) that robustly activates TLR4 signaling and NLRP3 inflammasomes. Yet bats thrive with this microbial composition without developing chronic inflammation. Bats further exhibit remarkably high intestinal permeability—L-rhamnose bioavailability up to 90% and >70% passive paracellular glucose absorption [41], compensating for reduced intestinal surface area required for flight. This combination of high Proteobacteria abundance, high permeability, and absent chronic inflammation represents a unique evolutionary solution, decoupling LPS exposure from inflammatory output.

Several bat-specific adaptations enable this tolerance. Constitutive endotoxin tolerance maintains immune cells in a controlled hyporesponsive state [42]. ASC2-mediated inflammasome suppression and reduced NLRP3 sensitivity prevent excessive LPS-triggered IL-1β release [31,32]. Constitutive IFN-α at epithelial barriers modulates bacterial product responses while maintaining immune vigilance [43,44]. Rapid gut transit (15–30 min vs. 24–48 h in humans) limits anaerobic niche formation and favors facultative anaerobes like *Proteobacteria* [39,45]. Together, these adaptations demonstrate how bats decouple microbial sensing from inflammatory execution. The bat microbiome contributes across multiple CLSV-6 dimensions: sub-inflammatory microbial exposure trains damage tolerance, microbial metabolites stimulate autophagy, chronic bacterial stimulation necessitates inflammasome dampening, and continuous microbial presence sustains basal immune readiness without overt inflammation [17,42,46].

*Proteobacteria* dominance in many bats is sustained by flight-specific physiological constraints absent in humans, in whom elevated Proteobacteria reliably predicts dysbiosis and systemic inflammation. The translational goal is instead to recapitulate the downstream anti-inflammatory outcomes bats achieve—dampened inflammasome activation, restrained LPS-driven cytokine responses, and maintained barrier integrity—within a human gut ecosystem optimally organized around Firmicutes/Bacteroidetes balance. Human dietary strategies, therefore, target the same functional endpoints through a biologically appropriate microbial route.

## 3. Human Longevity: Efficient Clearance Without Immune Escalation

Centenarian studies consistently associate human longevity with controlled inflammation and preserved immune competence rather than heightened immune activation, challenging the view that stronger immune responses ensure healthier aging [47,48,49].

### 3.1. Immune Characteristics of Long-Lived Individuals

Long-lived individuals tend to exhibit a distinctive inflammatory balance rather than simple immune suppression. While CRP, IL-6, and TNF-α are often elevated in centenarians compared with young adults, these increases are accompanied by proportional elevations in IL-10, transforming growth factor (TGF)-β1, and IL-1RA, creating an equilibrium that prevents unopposed inflammatory amplification [48,49,50]. Elevated IL-6 and CRP predict mortality at ages 65–80 but not beyond 90 years [8,51], confirming that single markers are context-dependent and that the relevant distinction lies in pro-/anti-inflammatory ratios and functional immune capacity rather than absolute levels. IL-6 and CRP may additionally participate in somatic maintenance and tissue repair rather than purely reflecting inflammation [8,52]. Centenarians maintain infection protection through preserved innate and adaptive immunity [49,53], retain natural killer (NK) cell activity and cytotoxic T cell potential [54], and show enhanced NK-T cell communication through major histocompatibility complex (MHC-I), cluster of differentiation (CD)-99, and macrophage migration inhibitory factor (MIF) pathways. Good immunity reflects proportionality, timing, resolution, and balanced cytotoxic capacity rather than persistent activation [54].

### 3.2. Enhanced Endogenous Damage Control

Long-lived individuals usually exhibit enhanced damage management across multiple systems. Autophagy is often preserved or enhanced in centenarians [55], with serum beclin-1 elevated 1.6-fold versus young controls [56], higher lysosomal activity in B lymphocytes, and preserved activation-induced autophagy in CD4+ T cells correlating with enhanced IFN-γ production—collectively limiting DAMP accumulation and chronic innate immune stimulation. Mitophagy prevents mtDNA and ROS release, both potent inflammasome activators [57].

Proteostasis networks appear to be more robust in centenarians [58]. Brazilian supercentenarians were shown to maintain proteasome activity at young-adult levels with upregulated autophagy-related gene (*ATG)-2A* and beclin 1 (*BECN1*), and proteodynamics approaching those of younger individuals except for mitochondrial ATP production [58]. DNA repair capacity is similarly improved [59]: centenarian fibroblasts showed low DNA damage, preserved telomeres, and high RNASEH2C levels that degrade cytoplasmic RNA–DNA hybrids before triggering interferon signaling—RNASEH2C knockdown increases IL-6 and IFN-β, confirming its causal anti-inflammatory role [60]. Rare variants in ATM and BRCA1 further support genomic stability [60]. Efficient senescent cell clearance is equally critical. Senescence drives chronic inflammation through senescence-associated secretory phenotype (SASP) factors, including IL-6, IL-8, IL-1β, TNF-α, CXCL9, and MMPs [60]. Centenarians tend to maintain enhanced cytotoxic CD4+/CD8+ T cells and elevated CD56+CD16+ NK cells with senolytic capacity [61,62], limiting SASP-mediated inflammation [63]. Perforin-deficient mice exhibit accelerated senescent cell accumulation, chronic inflammation, and shortened lifespan—conditions reversed by senolytics—supporting the causal importance of this clearance capacity [63].

### 3.3. Reframing Inflammaging

Inflammaging has been framed as a primary driver of aging [10], with some evidence suggesting it may reflect intrinsic immune dysregulation independent of upstream damage [3,64]. A complementary and well-supported interpretation holds that chronic inflammation arises, at least in part, as a secondary consequence of accumulated cellular damage [1]. When clearance systems become insufficient, danger signals—including oxidized lipids, protein aggregates, mitochondrial DNA (mtDNA), and SASP factors—persistently engage immune sensing, sustaining inflammatory tone. Under this model, chronic inflammation serves as both a secondary causal factor in age-related pathologies and a biomarker of impaired damage management—explaining why anti-inflammatory interventions targeting single cytokines often fail to extend healthspan [65]: they address a downstream signal without resolving the upstream burden that perpetuates it.

### 3.4. Evolutionary Constraints

Bats evolved under unique selective pressures: powered flight generates ~15–20 times resting metabolic output, imposing strong selection for minimizing inflammatory tissue damage under high metabolic stress [22,35], while colonial roosting and persistent viral exposure selected for tolerance-based immunity [66]. Humans evolved differently—for most of evolutionary history, mortality stemmed from infection, trauma, and reproductive risks rather than chronic degenerative disease [67], favoring rapid inflammatory responses despite long-term tissue costs [68]. Antagonistic pleiotropy predicts that alleles conferring robust early-life inflammatory responses enhance pathogen resistance but contribute to chronic low-grade inflammation and tissue degeneration with aging [69].

### 3.5. Cost of Hyperinflammation

Chronic inflammation contributes to cancer, metabolic disorders, cardiovascular disease, neurodegeneration, sarcopenia, osteoporosis, and functional decline. Mechanistically, persistent nuclear factor kappa-light-chain-enhancer of activated B (NF-κB) activation induces pro-inflammatory genes, sustained NLRP3 generates IL-1β/IL-18 [70,71], chronic IL-6 trans-signaling promotes insulin resistance and vascular inflammation [71], TNF-α drives endothelial dysfunction and muscle wasting [72,73], and SASP creates self-amplifying inflammatory loops [74]. Damage containment and resolution strategies are central to healthy aging [75,76].

### 3.6. Conceptual Model

Human longevity is associated with distinct peripheral immune cell signatures that differ markedly from typical aging trajectories [77], suggesting that immune competence is a defining feature of long-lived individuals. Immune resilience—the capacity to maintain balanced immune responses despite inflammatory stress—is progressively diminished in typical aging, and its preservation is associated with longevity and resistance to infection [78]. The centenarian phenotype—balanced mediators, preserved autophagy, enhanced proteostasis, efficient DNA repair, and effective senescent cell clearance—parallels bat strategies, suggesting that interventions enhancing damage management will outperform simple inflammation suppression.

## 4. Convergent Biological Principles Across Species

**Figure 2:**
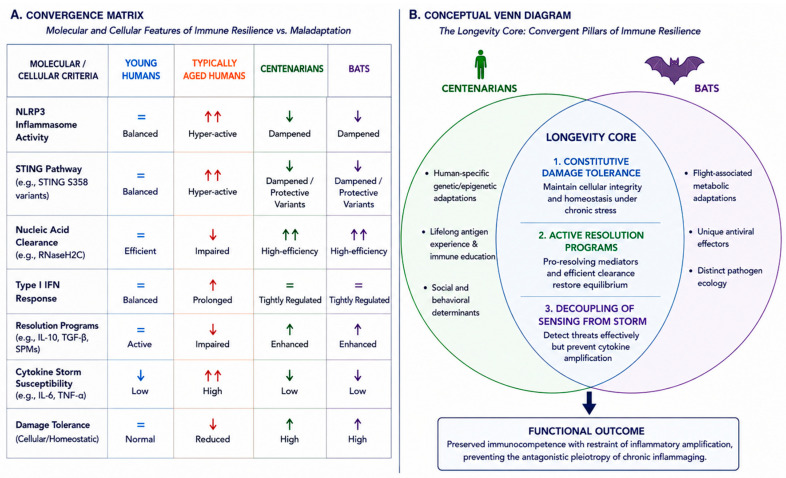
Convergent immunophysiology architectures across species. It summarizes the convergent biological principles that decouple immune sensing from inflammatory amplification in long-lived models. (**A**) Convergence Matrix: Specific molecular features are compared across young humans, typically aged humans, centenarians, and bats. While typically aged humans exhibit hyperactive NLRP3 signaling and impaired inflammatory resolution, bats and centenarians converge on high-efficiency nucleic acid clearance mechanisms, such as RNaseH2C activity, and dampened inflammasome triggers, such as STING S358 variants, thereby supporting the convergence hypothesis through shared molecular characteristics. (**B**) Conceptual Venn Diagram: Despite substantial phylogenetic distance, the “Longevity Core” shared by bats and centenarians is defined by three major pillars: constitutive damage tolerance, active resolution programs, and the decoupling of pathogen sensing from cytokine “storm” responses. This architecture preserves immunocompetence while preventing the antagonistic pleiotropy associated with chronic inflammaging. ↑↑: strongly increased or hyperactivated responses; ↑: moderate enhancement or activation; ↓: reduced or suppressed activity; =: maintenance of a stable or balanced state.

Comparative analyses reveal a unifying principle: longevity is promoted by decoupling immune sensing from inflammatory amplification rather than augmenting immune magnitude. In humans, immune activation against foreign or damaged self-materials typically culminates in persistent low-grade chronic inflammation [3]. Long-lived bats increase immune readiness without proportional inflammation [23], and long-lived humans converge toward this architecture, preserving immune competence while restraining inflammatory amplification [79]. This immune resilience—balancing immunocompetence with controlling inflammation—forms the conceptual core of this review (Figure 2) [54].

### 4.1. Damage Tolerance over Immune Resistance

Foreign material accumulation triggers immune resistance programs prioritizing elimination. While effective acutely, this becomes maladaptive with aging: incomplete clearance generates persistent danger signals, chronic activation, and tissue damage. Disease tolerance protects against physiological damage without reducing pathogen burden, whereas resistance eliminates pathogens through inflammatory mechanisms—strategies exhibiting antagonistic pleiotropy, with heightened early-life inflammatory responses conferring survival advantages but contributing to chronic inflammation and tissue degeneration later [80]. Bats prioritize damage tolerance over maximal resistance, sustaining constitutive antiviral defenses while limiting inflammatory effector responses [19]. Enhanced autophagy and DNA repair limit DAMP accumulation and inflammatory signaling [15,36], while dampened inflammasomes prevent chronic IL-1β/IL-18 secretion [81]. Centenarians mirror this strategy: efficient autophagy, preserved proteostasis, robust DNA repair, and balanced inflammatory profiles with proportional anti-inflammatory mediators [50,56,77]. Single-cell transcriptomics confirm enhanced immune resilience—preserved immunocompetence and controlled inflammation despite antigenic challenges [82,83].

### 4.2. Decoupling Sensing from Inflammatory Amplification

In typical humans, PRR activation—through TLRs, RLRs, NLRs, and interferon pathways—is tightly linked to inflammasome activation and cytokine amplification, converting immune surveillance into chronic tissue damage [84]. Long-lived bats selectively loosen this linkage through multiple adaptations [11,28,85]: the S358 STING mutation prevents DNA damage from triggering inflammation [26,27]; *PYHIN* gene family loss, including *AIM2*, eliminates DNA-sensing inflammasomes [28]; ASC2-mediated inhibition and NLRP3 variants dampen inflammasome activation [31]; constitutive IFN-α establishes basal antiviral readiness without chronic stress [11]; and TLR2 substitutions reduce inflammatory cytokines while preserving pathogen detection [57]. Sensing remains intact, priming preserved, but execution is restrained at multiple checkpoints [28].

Centenarians appear to exhibit parallel architecture. Preserved immune competence coexists with balanced mediator profiles [48,82,86], with cytotoxic capacity retained through elevated NK cells, enhanced CD4+ CTLs, preserved TCR diversity, and upregulated DNA repair and stress resistance programs. Immune resilience—quantifying the balance between longevity-associated immunocompetence and mortality-associated inflammation [78,87]—confers lower infection risk, superior survival during sepsis and COVID-19, and extended longevity [87]. Centenarians thus reflect reprogrammed immune–inflammation interfaces that successfully decouple sensing from amplification across a lifetime of antigenic exposure [4,42]. The shared molecular features defining this convergence are summarized in Table 2.

### 4.3. Rapid Resolution and Return to Homeostasis

Longevity depends more on how immune responses resolve than how strongly they initiate. Resolution is an active process mediated by specialized pro-resolving mediators (SPMs)—resolvins, protectins, and maresins—that terminate neutrophil recruitment, promote efferocytosis, and restore homeostasis [33,88]. Bats achieve rapid resolution through elevated autophagy and mitophagy that continuously remove danger signals before chronic inflammation is triggered [33], efficient mitophagy preventing mtDNA and ROS release [25], and constitutive IL-10 promoting immune contraction [89]. Centenarians display the same phenotype: preserved autophagy, reduced senescent burden through cytotoxic surveillance, elevated RNASEH2C degrading RNA–DNA hybrids before triggering interferon signaling [60], and enhanced NK/CTL populations eliminating senescent cells before SASP amplification [62]. Perforin-deficient mice show accelerated senescence, chronic inflammation, and shortened lifespan—reversed by senolytics—confirming the causal importance of cytotoxic resolution capacity [90].

Typical aging is characterized by resolution failure: declining autophagy accumulates aggregates and damaged mitochondria, defective clearance of senescent cells permits self-amplifying SASP loops, and inadequate SPM production prevents inflammatory termination [88]. Together, these convergent principles explain why bat immunity provides a human longevity blueprint—high immune readiness coupled with restrained inflammation and efficient resolution [32]—with shared mechanisms across phylogenetically distant mammals suggesting evolutionarily conserved strategies for inflammation-resistant survival.

## 5. Core Longevity State Vector (CLSV-6): A Multidimensional Immunotype Framework

**Figure 3:**
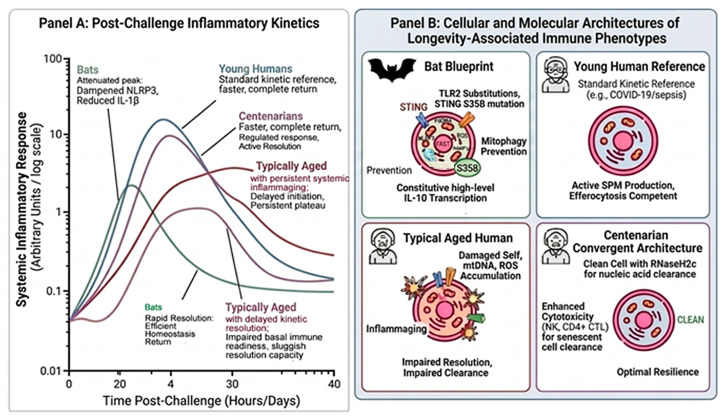
Cellular and molecular architectures of longevity-associated immune phenotypes. This integrative figure demonstrates how the six dimensions of the Core Longevity State Vector (CLSV-6) determine systemic inflammatory outcomes. (**A**) Post-Challenge Kinetics: High-resilience phenotypes (Bats, green; Centenarians, purple) exhibit “Optimal Resilience,” characterized by efficient initiation followed by rapid, complete resolution to the homeostasis baseline. This contrasts with the “Maladaptive Plateau” seen in typical human aging (Red), where resolution failure leads to chronic inflammaging. (**B**) Cellular Architecture: The underlying molecular drivers of these curves include decoupled sensing, such as STING S358 variants in bats, and enhanced cytotoxic surveillance, such as NK and CD4+ CTL maintenance in centenarians. These mechanisms ensure that “Damage Tolerance” and “Rapid Resolution” remain the dominant physiological states throughout the lifespan.

Biological longevity is not the result of a single genetic “switch” but emerges from a highly coordinated physiological configuration that integrates acute immune readiness with long-term damage management [91]. To formalize this, we propose the Core Longevity State Vector (CLSV-6)—a multidimensional framework that quantifies the balance between inflammatory sensing, cytotoxic resolution, and tissue tolerance (Table 3). This vector explains the “longevity paradox” seen in phylogenetically distant models: how bats achieve extreme lifespans despite high metabolic rates and persistent viral exposure, and why centenarians converge on a nearly identical immunophysiological architecture [92]. As illustrated in Figure 3, the CLSV-6 dimensions translate into observable kinetic differences. While typical human aging is characterized by a “failure plateau” where inflammatory signals decouple from resolution programs, the high-resilience models (bats and centenarians) maintain a tight linkage between sensing and rapid homeostasis [58]. By grounding the CLSV-6 in shared molecular features—such as dampened NLRP3 activity and optimized nucleic acid clearance—this framework moves beyond descriptive aging models [37]. It provides a falsifiable basis for identifying “optimal immunotypes,” suggesting that extreme survival depends less on the magnitude of the immune response and more on the precision of its termination.

### 5.1. Why Single Biomarkers Fail

IL-6 and CRP predict mortality in individuals aged 65–85 but not in centenarians, in whom elevated levels coexist with proportionally elevated anti-inflammatory mediators [6]. Single biomarkers cannot capture the multidimensional nature of biological aging [93]: centenarians display coordinated patterns across metabolic, inflammatory, liver, and renal markers rather than single exceptional values [94]. IFN signaling reflects antiviral competence but cannot distinguish protective readiness from pathological escalation—bats maintain constitutive IFN-α without inflammation, whereas persistent IFN in humans causes autoimmune pathology [95]. Aging affects organ systems at different rates [96], and attributing longevity to single markers fails to capture emergent properties arising from their coordination [97].

### 5.2. Longevity as a Coordinated State

A cross-species transcriptomic analysis of 43 mammals confirmed that longevity mechanisms connect across hallmarks, with upregulation of DNA repair and downregulation of protein degradation in long-lived species [98]. Long-lived bats tend to integrate enhanced DNA repair, dampened STING [27], elevated autophagy [12], and restrained inflammasomes as a system [28]. Centenarians exhibit balanced inflammation [48], preserved immune competence [83], efficient autophagy [58,99], maintained proteostasis, improved DNA repair [60], and reduced senescent burden [62,100,101]—collectively representing immune resilience [82,83,101].

### 5.3. CLSV-6: Six Core Dimensions

Dimension 1: Damage tolerance operates upstream of immune activation, preventing damage perception as a persistent threat [83,102]. Bats typically show enhanced DNA repair, an S358 STING mutation that prevents inflammatory cascades [19], and PYHIN loss, eliminating DNA-sensing inflammasomes [28]. Centenarians exhibit low DNA damage, preserved telomeres [103], and high RNASEH2C levels, which degrade RNA–DNA hybrids before triggering interferon [60]. *RNaseH2C* knockdown increases IL-6/IFN-β, confirming causality [60].

Dimension 2: Autophagy–Mitophagy removes DAMPs before inflammatory thresholds are reached [104]. Bats exhibit elevated LC3-II/LC3-I (microtubule-associated protein 1 light chain 3) ratios [105] and signatures of positive selection in multiple autophagy-related genes [34], limiting mt DNA and ROS release [34,106]. Compared with non-centenarian older adults, centenarians exhibit elevated serum beclin-1 levels (2.2 vs. 1.4 ng/mL) [106], increased lysosomal activity [107], and preserved activation-induced autophagy associated with interferon-γ production [99]. Consistently, *ATG5* overexpression extends lifespan and health span in mice.

Dimension 3: Proteostasis limits aggregate-driven inflammation [108]. Centenarians have been shown to maintain proteasome activity at young-adult levels [109], show *ATG2A/BECN1* upregulation [104], and achieve proteodynamics approaching those of the young, except for mitochondrial ATP [58]. Cross-species analysis links proteostasis maintenance to longevity [35,98].

Dimension 4: Basal immune readiness decouples surveillance from amplification [85,110]. Bats constitutively express interferon-stimulated genes (ISGs) and IFN-α while avoiding inflammatory stress through downstream dampening [11,43,111]. Centenarians retain cytotoxic capacity (elevated NK cells, enhanced CD4+ CTLs), preserved TCR diversity [83,112], and increased gene expression associated with immune response, alongside DNA repair programs [82,83,86,112].

Dimension 5: Inflammasome regulation limits inflammatory effector responses while preserving immune sensing [26,27,31,32,113]. Bat NLRP3 contains substitutions reducing priming responsiveness [31], and ASC2 acts as a dominant-negative inhibitor [32]. Centenarians balance pro- and anti-inflammatory cytokines [48], raising thresholds for DAMP-triggered responses [3,48,85,113]. ASC2 reduces inflammation in mouse disease models [32].

Dimension 6: Resolution capacity actively terminates activation via SPMs, including resolvins, protectins, and maresins [88,114]. Bats use mitophagy [33,105], DNA repair [88,106], and IL-10 [89] for rapid return to homeostasis [105]. Centenarians maintain autophagy [56,99], cytotoxic senescent cell surveillance [62,100], elevated NK/CTL populations that prevent SASP [62,115], and preserved SPM pathways [88,114,116]. Perforin-deficient mice show accelerated senescence and shortened lifespan, which are reversed by senolytics [90,115]. Typical aging represents resolution failure: declining autophagy, defective clearance enabling SASP loops, and inadequate SPM production [88].

### 5.4. CLSV-6 Immunotype Framework: Integrated Concept

Bats occupy an optimal multidimensional state, achieving exceptional longevity through coordinated regulation across all six CLSV dimensions despite intense metabolic demands and chronic viral exposure [22,45]. Centenarians partially converge toward this configuration, suggesting that healthy human longevity represents an incomplete but biologically recognizable realization of bat-like immunophysiological architecture [82,91]. The CLSV-6 framework explains why single biomarkers often fail to predict healthy aging, as isolated measurements cannot capture emergent properties arising from system-level coordination [117,118]. Accordingly, the framework provides an operational model for healthy aging that can guide biomarker development, intervention design, and translational research [93,119,120]. Rather than targeting individual pathways in isolation, effective interventions may need to enhance coordinated function across all six dimensions, thereby shifting the system toward the longevity-associated configuration observed in bats and centenarians (Table 4). At the molecular level, bats maintain antiviral protection through constitutive interferon-α signaling, restrained STING and NLRP3 inflammasome activation, enhanced autophagy–mitophagy, and efficient DNA repair mechanisms. Centenarians also exhibit parallel preservation of interferon readiness, proteostasis, autophagic competence, and inflammatory resolution pathways. Together, these features support cross-species convergence toward inflammation-resistant aging.

## 6. Mechanisms of Foreign Material Management Without Hyperinflammation

This section details the cellular and molecular machinery that executes this state, focusing on pathways that enable efficient handling of foreign and damage-associated materials without triggering chronic inflammation.

### 6.1. Foreign and Danger-Associated Materials Driving Immune Aging

Foreign materials that chronically engage the immune system during aging fall into two broad classes: pathogen-associated molecular patterns (PAMPs) and DAMPs [121]. PAMPs—viral RNA and DNA, bacterial LPS, peptidoglycans, and fungal β-glucans—activate TLRs, retinoic acid-inducible gene (RIG)-I-like receptors, NOD-like receptors, and cGAS, sustaining innate immune signaling even without overt infection [121,122]. Persistent exposure to gut commensal products and chronic viral infections, including cytomegalovirus (CMV), Epstein–Barr virus (EBV), and herpes simplex virus (HSV), maintains continuous PRR engagement.

DAMPs arise from aging itself [121,123]. Nucleic acid DAMPs—oxidized DNA, cytosolic mtDNA, self-RNA, and RNA–DNA hybrids—activate cGAS-STING, TLR9, RIG-like receptors (RLRs), and AIM2 inflammasomes. Protein DAMPs, including HMGB1, HSPs, S100 proteins, histones, and serum amyloid A, activate NF-κB via TLR2/4/9 and RAGE, with HSP70 prompting TNF-α and IL-6 secretion [121]. Lipid and metabolic DAMPs—advanced glycated end product (AGEs), lipid peroxidation products, oxidized cardiolipin, and cholesterol crystals—activate the TLR4-MD-2 complex and directly bind NLRP3 [121]. Purine metabolites, including extracellular ATP, uric acid crystals, and hyaluronan fragments, stimulate NLRP3, activating caspase-1 and inducing IL-1β/IL-18 release. Accumulation of senescent cells releasing SASP, damaged mitochondria leaking mtDNA, and oxidative stress generating protein modifications creates a persistent DAMP burden sustaining low-grade inflammation [124,125]. Bats and long-lived humans differ not in the absence of these materials but in how effectively they contain and clear them before driving inflammatory amplification [123,126].

### 6.2. Non-Inflammatory Clearance Pathways

Longevity depends on clearance systems removing foreign and damaged materials while minimizing immune activation [101]. These pathways function upstream of inflammatory signaling, preventing DAMP accumulation from reaching immunogenic thresholds. Autophagy eliminates damaged proteins, viral components, and oxidized macromolecules before they reach immunogenic thresholds [101,127]. Bats show elevated basal autophagy limiting cytosolic DNA and mitochondrial debris, with age-related LC3-II/I increases absent in mice [105]; ATG5 overexpression extends lifespan in mice while deficiency accelerates aging. Mitophagy removes dysfunctional mitochondria, preventing mtDNA and ROS release that drive NLRP3 activation [34,106]; bats maintain efficient mitophagy through enhanced PTEN-induced kinase 1 (PINK1) and Parkin expression [33,105], and preserved mitophagy associates with human longevity, while impairment contributes to neurodegeneration and metabolic dysfunction [128].

The ubiquitin-proteasome system limits misfolded protein accumulation that activates innate immune pathways [108]. Supercentenarians maintain proteasome activity at young-adult levels [109], and HSP70/90-mediated heat shock responses prevent protein misfolding, with long-lived species showing enhanced chaperone and proteasome expression [37]. Efferocytosis—macrophage clearance of apoptotic and senescent cells—prevents secondary necrosis and DAMP release [129]; without rapid clearance, apoptotic cells release HMGB1, DNA, histones, and DAMPs, triggering robust inflammation. Efficient efferocytosis additionally promotes SPM production [88,130]. Aging impairs efferocytosis through reduced T-cell/transmembrane immunoglobulin and mucin domain containing 4 (TIM-4) expression via elevated p38 mitogen-activated protein kinases (MAPK) [131,132], while senescent cells upregulate CD47 “don’t-eat-me” signals. Centenarians maintain efficient efferocytosis through elevated NK cells and CMV-specific cytotoxic T lymphocytes (CTLs) with senolytic capacity, preventing SASP-mediated inflammation [62,90,100,115]. Together, these pathways form CLSV-6′s cellular execution layer, removing damage-derived materials before chronic immune activation.

### 6.3. Decoupling Clearance from Immune Amplification

Bat immunity and human longevity share a defining feature: clearance is not obligatorily linked to inflammatory amplification [28,105]. In typical aging, cytosolic DNA and mitochondrial debris accumulation activate cGAS-STING and NLRP3, driving sustained IL-1β, IL-6, and TNF-α production, with inflammasome thresholds decreasing and response magnitude increasing with age [133]. As clearance capacity declines, DAMPs persist, sustaining chronic activation.

Long-lived bats tune sensing pathways so that DNA detection supports antiviral defense while inflammasome activation is restrained [31,32]. The S358 STING mutation prevents endogenous DNA from triggering inflammation [26,27], PYHIN family loss eliminates DNA-sensing inflammasomes [134], ASC2-mediated NLRP3 suppression uncouples priming from execution [32], and constitutive IFN-α maintains antiviral readiness without chronic inflammation [43,111]. Enhanced autophagy and mitophagy continuously clear DAMPs before inflammatory thresholds are reached [24,98]. Long-lived humans exhibit parallel phenotypes: sensing remains intact, but amplification is limited by enhanced autophagy [99], elevated RNASEH2C degrading RNA–DNA hybrids [60], effective senescent cell clearance [62,115], balanced pro- and anti-inflammatory mediators [48], and elevated IL-10 and TGF-β promoting resolution [88].

Mechanistically, longevity prioritizes clearance and resolution upstream of inflammation: damage is removed before becoming immunogenic [101,135], sensing does not escalate into sustained cytokine production due to inflammasome regulation and dampened STING signaling [31], and homeostasis is rapidly restored through efferocytosis, SPM production, and active resolution pathways [26,88,129]. This architecture explains how bats and centenarians maintain high immune readiness without hyperinflammation and provides a mechanistic foundation for nutritional interventions targeting clearance, regulation, and resolution without immune overstimulation [101,120].

## 7. Nutritional and Functional Food Interventions: CLSV-6–Aligned Strategy

### 7.1. Biological Plausibility of Nutritional Modulation

**Figure 4:**
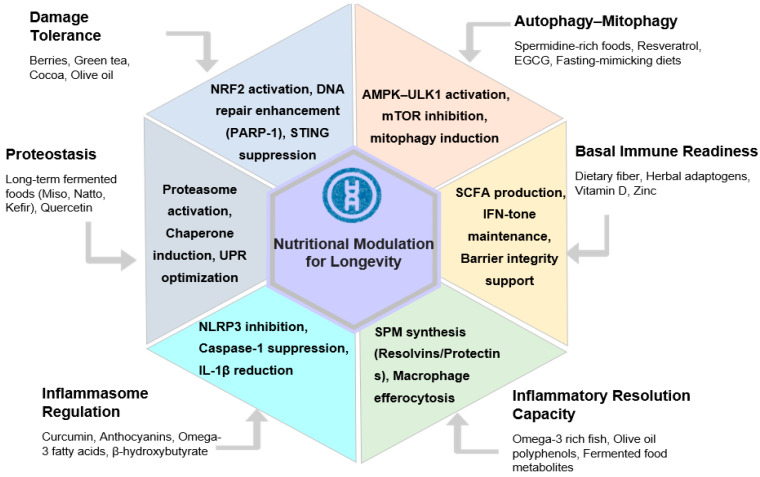
Nutritional strategies targeting the Core Longevity State Vector (CLSV-6) for human healthspan enhancement. This multidimensional framework summarizes the nutritional strategies required to transition human physiology toward a “bat-like” resilient state. (1) Mechanistic Integration: the central hexagon identifies six core domains—Damage Tolerance, Autophagy–Mitophagy, Proteostasis, Resolution Capacity, Inflammasome Restraint, and Basal Immune Readiness. (2) Targeted Bioactivities: specific functional foods and compounds (e.g., spermidine, quercetin, omega-3 fatty acids, and fermented foods) are mapped to their primary molecular targets, such as NRF2, AMPK, and NLRP3. (3) Physiological Outcome: systematic modulation of these domains aims to reduce inflammaging and improve systemic resilience, ultimately promoting healthy human longevity.

Nutritional modulation offers a biologically coherent strategy for shifting humans toward CLSV-6 longevity states. Dietary exposures operate at low intensity, engage multiple convergent pathways simultaneously, and can be sustained over decades with minimal toxicity compared with pharmacological interventions [136,137]. Functional foods integrate across the six longevity mechanisms by supporting cellular damage handling, immune surveillance, inflammasome restraint, and inflammatory resolution rather than chronic immune activation (Figure 4; Table 5). Importantly, many of the described biological effects have been observed in humans at physiologically achievable dietary or supplemental intake ranges, although responsiveness varies substantially according to microbiome composition, genetics, baseline inflammatory status, and long-term dietary adherence. Rather than producing categorical immune suppression, these interventions are proposed to induce gradual systems-level shifts toward inflammation-resistant immune regulation through cumulative microbiota-mediated and cell-intrinsic mechanisms.

Polyphenol-rich foods—including berries, green tea, cocoa, and olive oil—enhance cellular resistance to oxidative and genotoxic stress through NRF2 and sirtuin pathway activation, stimulation of DNA repair pathways, and suppression of cytosolic DNA sensing and cGAS–STING signaling [138]. Key polyphenols such as resveratrol, epigallocatechin gallate (EGCG), curcumin, and quercetin activate AMP-activated protein kinase (AMPK) while inhibiting mammalian target of rapamycin (mTOR), thereby promoting autophagy, mitochondrial quality control, and clearance of damaged proteins and organelles [139]. These mechanisms are particularly relevant in senescent cells, where cytoplasmic chromatin fragments and mitochondrial DNA activate cGAS–STING and NLRP3 signaling, amplifying sterile inflammation and the senescence-associated secretory phenotype (SASP). Several polyphenols and omega-3 fatty acids attenuate this process by reducing oxidative DNA damage, enhancing mitophagy, and suppressing NF-κB-, caspase-1-, and IL-1β-mediated inflammatory amplification [140]. Large cohort studies associate higher polyphenol intake with reduced all-cause mortality and lower incidence of cardiometabolic and neurodegenerative diseases [140,141].

A critical translational caveat is that many mechanistic findings derive from in vitro studies using concentrations substantially exceeding those achievable through conventional dietary intake. Resveratrol, curcumin, and EGCG exhibit limited oral bioavailability because of rapid first-pass metabolism, poor intestinal absorption, and chemical instability. However, gut microbial biotransformation partially compensates for these limitations by converting parent polyphenols into bioactive metabolites—including urolithins from ellagitannins, equol from daidzein, and hydroxycinnamic acid derivatives from chlorogenic acids—that are more readily absorbed and retain biological activity at physiologically relevant concentrations. These microbial metabolites likely mediate a substantial proportion of polyphenol-associated health effects, and their production is highly microbiome-dependent, potentially explaining marked interindividual variability in responsiveness. Translational strategies should therefore evaluate metabolite profiles and microbiome composition in addition to parent compound intake.

Caloric restriction mimetics and plant-derived bioactive compounds—including resveratrol, spermidine, quercetin, and EGCG—further activate the AMPK–ULK1 axis while suppressing mTOR signaling, thereby increasing autophagic flux and mitochondrial quality control [142]. In the 20-year Bruneck Study (*n* = 829), higher dietary spermidine intake was associated with lower all-cause, cardiovascular, and cancer-related mortality. Resveratrol and epicatechin additionally stimulate mitochondrial biogenesis through PGC-1α signaling, promoting mitophagy and limiting oxidative stress accumulation [143]. Polyphenols and fermented foods also enhance chaperone networks, proteasome activity, and unfolded protein response efficiency, thereby reducing accumulation of misfolded proteins that can trigger innate immune activation and neurodegenerative processes [144]. Traditional fermented foods—including miso, doenjang, kefir, and natto—have been associated with reduced frailty and cardiometabolic risk [145,146], consistent with experimental evidence supporting improved proteostatic resilience.

Importantly, human nutritional strategies are not intended to replicate bat gut microbial architecture, which is frequently enriched in Proteobacteria under flight-associated physiological constraints, but rather to reproduce downstream anti-inflammatory and resilience-associated outcomes within the human intestinal ecosystem organized around Firmicutes/Bacteroidetes-dominated communities. Dietary fiber, fermented foods, and herbal adaptogens may support this objective by maintaining antiviral competence while lowering chronic inflammatory tone. Short-chain fatty acids (SCFAs) and indole derivatives generated through microbial fermentation enhance interferon signaling and barrier immunity while restraining cytokine amplification. Butyrate suppresses histone deacetylase (HDAC) and NF-κB activity in macrophages, while signaling through FFAR2/GPR109A promotes IL-18 production, intestinal integrity, and regulatory T-cell differentiation. A 17-week randomized dietary intervention demonstrated that high-fermented-food intake increased microbiome diversity while reducing inflammatory markers [147], supporting immune preparedness without persistent elevation of IL-6 or CRP.

Polyphenols, omega-3 fatty acids, and fermented-food-derived metabolites additionally suppress NLRP3 inflammasome activation without abolishing pathogen sensing [148]. Quercetin and fisetin inhibit PI3K/AKT/mTOR signaling and inflammatory cytokine secretion in LPS-stimulated macrophages, whereas EGCG suppresses mTOR–HIF-1α signaling. Human intervention studies consistently report reductions in CRP and IL-1β following omega-3 fatty acid intake [149,150]. Eicosapentaenoic acid (EPA) and docosahexaenoic acid (DHA) reduce NLRP3 activation through membrane phospholipid remodeling and lipid raft disruption while also serving as substrates for specialized pro-resolving mediators (SPMs), including resolvins, protectins, and maresins [88,151]. Polyphenols and fermented-food metabolites further enhance macrophage efferocytosis, mitophagy, and immune contraction [152], whereas SCFAs—particularly butyrate and propionate—strengthen intestinal barrier integrity through tight-junction upregulation mediated by NLRP3 regulation, autophagy modulation, FFAR signaling, and HDAC inhibition. A Mediterranean diet supplemented with olive oil or nuts reduced major cardiovascular events [153]. Collectively, these findings support the concept that nutrition promotes damage management and inflammatory resolution rather than nonspecific immune stimulation, partially recapitulating physiological characteristics shared by bats and exceptionally healthy aging humans [120].

### 7.2. Evidence Convergence on CLSV-6

Functional food research converges across all six CLSV-6 dimensions, although these effects have traditionally been studied as isolated mechanisms rather than components of an integrated longevity architecture. Polyphenols—including resveratrol, quercetin, EGCG, and curcumin—enhance damage tolerance and proteostasis through NRF2 and SIRT1 activation [138], induce autophagy–mitophagy via AMPK–mTOR modulation, restrain cGAS–STING/NLRP3 inflammatory amplification associated with senescent-cell signaling, and promote inflammatory resolution through specialized pro-resolving mediator biosynthesis [154]. Fermented foods—including miso, doenjang, kimchi, kefir, and natto—support basal immune readiness through enhancement of interferon signaling and mucosal defense [155], promote autophagy–mitophagy and inflammatory resolution through SCFA and indole production [156], and restrain inflammasome amplification via postbiotic peptides and microbial metabolites [157]. Butyrate rescues colonocytes from mitochondrial respiration and autophagy deficits [158], while SCFA production itself depends strongly on dietary fiber availability; under low-fiber conditions, microbial metabolism shifts toward amino acid and fat utilization, reducing SCFA generation [159]. Herbal extracts—including ginseng, turmeric, astragalus, and green tea—further reinforce damage tolerance and proteostasis through sirtuin and NRF2 signaling, stimulate autophagy–mitophagy through ginsenosides and catechins, and enhance inflammatory resolution through lipid mediator class switching and macrophage efferocytosis. Collectively, these functional foods converge toward a CLSV-6-associated physiological configuration characterized by reduced immunogenic burden, enhanced cellular clearance, restrained inflammatory amplification, and strengthened inflammatory resolution [160]. Herbal extracts—including ginseng, turmeric, astragalus, and green tea—further reinforce damage tolerance and proteostasis through sirtuin and NRF2 signaling, stimulate autophagy–mitophagy through ginsenosides and catechins, and enhance inflammatory resolution through lipid mediator class switching and macrophage efferocytosis. Collectively, these functional foods converge toward a CLSV-6-associated physiological configuration characterized by reduced immunogenic burden, enhanced cellular clearance, restrained inflammatory amplification, and strengthened inflammatory resolution

## 8. Translational Challenges and Future Directions

### 8.1. Challenges in Translation

The CLSV-6 framework provides a translational model for developing human longevity interventions that enhance immune resilience while limiting chronic inflammatory burden. The translational relevance of this framework is supported by evidence that centenarians naturally exhibit several immunophysiological characteristics convergent with bat longevity biology, including restrained inflammaging, preserved interferon responsiveness, enhanced autophagic competence, and improved inflammatory resolution. Thus, the translational objective is not to reproduce bat immunity in humans, but to strengthen resilience-associated pathways already observed in exceptionally healthy aging humans. Nevertheless, several biological and clinical challenges must be addressed before broad implementation.

Excessive immune suppression must be avoided, as healthy longevity depends on preserving basal immune readiness while restraining inflammatory amplification, thereby promoting immune resilience rather than immunosuppression [83,112]. Polyphenols at physiological doses can support immune competence by enhancing T-cell proliferation, NK cell activity, and interferon responses [161]. A fundamental biological asymmetry must also be acknowledged when translating bat immune adaptations into human dietary strategies. Bat immune tolerance is rooted in fixed germline evolution: the S358 STING mutation, *PYHIN* gene family loss, and *ASC2* expression represent permanent, high-magnitude genomic adaptations shaped over evolutionary timescales. By contrast, dietary modulation of STING signaling through polyphenols or omega-3 fatty acids operates through transient, dose-dependent, and substantially weaker mechanisms. Functional foods may therefore shift inflammatory thresholds incrementally toward a more inflammation-resistant physiological state, but cannot reproduce the categorical immune adaptations observed in bats.

Interindividual variability driven by microbiome composition, genetics, and life-course exposures strongly influences responsiveness to dietary interventions, necessitating precision-nutrition approaches [162]. A high-fiber intervention study identified three distinct immunological trajectories associated with baseline microbiota diversity [147], underscoring the importance of characterizing individual microbiota composition, SCFA production capacity, and baseline inflammatory status. Actionable biomarkers for autophagic flux, inflammasome regulation, and inflammatory resolution remain underdeveloped, although emerging candidates—including serum beclin-1 for autophagy, IL-1β/IL-10 ratio for inflammasome regulation [48], and specialized pro-resolving mediator (SPM) profiling for resolution capacity [163]—warrant longitudinal validation. Establishing optimal dose–response relationships also remains challenging because epidemiological studies generally report benefits at dietary intake levels, whereas mechanistic studies often employ supraphysiological concentrations. Furthermore, the hormesis of polyphenols necessitate careful dose selection. Long-term adherence additionally requires consideration of cultural acceptability, palatability, cost, and accessibility.

Several proposed CLSV-6 biomarkers—including circulating beclin-1, SPM profiles, and LC3-II/I ratio—are not yet standardized for routine clinical implementation. Beclin-1 ELISA assays remain largely research-grade with limited interlaboratory reproducibility; SPM profiling requires specialized lipidomic platforms; and LC3-II/I measurement in peripheral blood is technically challenging. By contrast, hs-CRP, IL-1β/IL-10 ratio, and vitamin D status are clinically feasible biomarkers with established reference ranges. Accordingly, the CLSV-6 biomarker panel should currently be regarded as a translational research framework requiring prospective validation before widespread clinical application. Despite these limitations, the CLSV-6 framework provides a clinically relevant foundation for integrating nutrition, immunology, microbiome science, and aging biology into a unified precision-longevity strategy. Several framework components—including dietary patterns, microbiome-targeted interventions, inflammatory biomarkers, and resolution-supporting nutritional approaches—are already compatible with existing preventive medicine and lifestyle intervention paradigms. Future longitudinal and interventional studies will determine whether coordinated modulation of CLSV-6 dimensions can improve healthspan, resilience to chronic disease, and healthy aging trajectories in humans.

### 8.2. Future Directions

The path forward demands integration of geroscience principles into clinical practice through developing and validating CLSV-6 biomarker panels for patient stratification, characterizing microbiome-dependent responder phenotypes, establishing evidence-based dose–response relationships for functional food interventions, conducting mechanism-driven clinical trials with healthspan rather than disease-specific endpoints, and implementing life-course preventive strategies targeting biological aging before multimorbidity onset. Bats achieved inflammation-resistant longevity through millions of years of selection under extreme metabolic and viral stress [22,45]. Humans, lacking such evolutionary adaptations, require deliberate interventions to recapitulate this state [83]. The CLSV-6 framework provides both the conceptual foundation and operational blueprint for this endeavor.

## 9. Conclusions

Healthy longevity depends on immune quality rather than intensity—the efficient management of biological damage without chronic inflammation. Bats achieve extreme lifespans through the coordinated action of six mechanisms: damage tolerance, autophagy–mitophagy, proteostasis, basal immune readiness, inflammasome restraint, and resolution capacity. Centenarians tend to converge toward this configuration, maintaining immune competence while balancing inflammatory mediators, achieving immune resilience without hyperinflammation. However, even centenarians are a diverse group, representing a wide range of ethnic backgrounds and lifestyles, and this variability is a confounding factor in drawing firm conclusions about the role of diet and lifestyle. Nevertheless, the CLSV-6 framework reframes longevity as the coordinated management of damage. Single biomarkers fail because they measure isolated dimensions; longevity emerges from the integrated function of all six mechanisms. Functional foods—polyphenols, fermented foods, omega-3 fatty acids, and caloric restriction mimetics—offer a practical translational strategy that converges on CLSV-6 by enhancing autophagy, restraining inflammasomes, promoting resolution, and maintaining proteostasis. Bats achieved inflammation-resistant longevity through millions of years of selection. Humans require deliberate intervention, and it is difficult to obtain long-term compliance with major dietary changes. By shifting the focus from immune stimulation to damage management, we can engineer a healthier form of human aging inspired by nature’s most successful longevity strategy.

## 10. Methods

This narrative review synthesizes evidence from bat immunology, centenarian research, and nutritional geroscience. We searched PubMed, Web of Science, and Google Scholar (inception through December 2025) using terms combining “bat” and “chiroptera” with immunity-related keywords; “centenarian”/“exceptional longevity” with inflammatory and cellular quality control markers; and “functional foods”, “polyphenols”, and “fermented foods” with aging mechanisms. We prioritized primary mechanistic studies, multi-omics investigations, and clinical trials, focusing on papers published during 2015 and 2025, alongside foundational work. Evidence was organized into six domains comprising the CLSV-6, with cross-species convergence identified when similar mechanisms appeared independently in bats and long-lived humans.

## Figures and Tables

**Table 1:** Examples of bats and comparable species of similar weight.

Species Name	Average Lifespan (Years)	Body Weight (g)	Typical Diet
Brandt’s Bat (*Myotis brandtii*)	41	7–9	Insects (beetles, moths)
Greater Mouse-Eared Bat (*Myotis myotis*)	37	28–40	Insects (beetles, grasshoppers)
Little Brown Bat (*Myotis lucifugus*)	6–34	8–11	Insects (midges, beetles, caddisflies)
Common Pipistrelle (*Pipistrellus pipistrellus*)	4–11	3.5–8.5	Insects (flies, moths)
Egyptian Fruit Bat (*Rousettus aegyptiacus*)	22	80–170	Fruit, nectar
Big Brown Bat (*Eptesicus fuscus*)	19	14–21	Insects (beetles, wasps)
Hoary Bat (*Lasiurus cinereus*)	6–7	20–35	Insects (moths, beetles)
Mexican Free-Tailed Bat (*Tadarida brasiliensis*)	8–10	11–12	Insects (moths, beetles, wasps)

**Table 2:** Mechanistic convergence matrix: shared molecular features of longevity-associated immunotypes.

CLSV-6 Dimension	Molecular /Cellular Marker	Typical Aging	Bat Convergence	Centenarian Convergence
Damage Tolerance	STING/DNA Sensing	Weaker	High/Rapid	High/Rapid
Autophagy–Mitophagy	Clearance Rate	Decreased	Enhanced /Rapid	Enhanced /Rapid
Proteostasis	RNaseH2C/DNA Repair	Vulnerable	Strong (PYHIN Loss)	Strong (RNaseH2C)
Basal Immune Readiness	IFN-α/PRR Priming	Reactive /Vulnerable	Constant /Strong	Preserved /Strong
Inflammasome Regulation	NLRP3/IL-1β Activity	Weak /Overactive	Restrained /Efficient	Restrained /Efficient
Inflammatory Resolution Capacity	Efferocytosis/SPMs	Weak /Overactive	Strong /Efficient	Strong/Efficient

SPMs, specialized pro-resolving mediators; STING, stimulator of interferon genes; RNASEH2C, ribonuclease H2 subunit C; PRR, pattern recognition receptors; IFN-α, interferone-α; NLRP3, NOD-like receptor pyrin domain-containing protein 3; IL-1β, interleukin-1β; PYHIN, pyrin and HIN domain family member 1.

**Table 3:** Core Longevity State Vector (CLSV-6): mechanistic domains and biological functions.

CLSV-6 Domain	Definition	Key Molecular Pathways	Functional Outcome (The “Tolerant & Clear” Goal)
Damage Tolerance	The capacity to withstand molecular damage and DAMPs without triggering inflammatory escalation.	DNA repair, NRF2, PARP, TP53 tuning	Reduced Danger Signaling: Prevents the immune system from “overreacting” to metabolic byproducts.
Autophagy–Mitophagy	Rapid removal and recycling of damaged organelles (mitochondria) and macromolecules.	AMPK–ULK1, PINK1–Parkin, TFEB	Mitochondrial Quality Control: Eliminates leaked mtDNA before it activates the inflammasome.
Proteostasis	Maintenance of protein folding, trafficking, and degradation systems to prevent aggregation.	UPR (Unfolded Protein Response), Proteasome, Chaperones	Reduced Proteotoxic Stress: Limits the accumulation of misfolded proteins that drive sterile inflammation.
Basal Immune Readiness	A “constantly primed” antiviral state that prevents viral replication without needing active inflammation.	Constitutive IFN (Interferon), Barrier Immunity	Rapid Pathogen Control: Enables immediate response to threats without the “cost” of a cytokine storm.
Inflammasome Regulation	High-threshold suppression of inflammatory amplification despite the presence of triggers.	NLRP3, STING, Caspase-1 regulation	Decoupled Sensing: Allows the body to “sense” a threat but “restrain” the inflammatory fire.
Inflammatory Resolution Capacity	The active biochemical process of terminating inflammation and initiating repair.	SPMs (Specialized Pro-resolving Mediators), Efferocytosis, Tregs	Tissue Recovery: Ensures inflammation is a temporary event, not a chronic state, leading to faster healing.

NRF2, nuclear factor erythroid 2–related factor 2; PARP, poly(ADP-ribose) polymerase; TP53, tumor protein p53; AMPK, AMP-activated protein kinase; ULK1, Unc-51-like autophagy activating kinase 1; PINK1, PTEN-induced kinase 1; TFEB, transcription factor EB; UPR, unfolded protein response; IFN, interferon; STING, stimulator of interferon genes; NLRP3, NOD-like receptor family pyrin domain containing 3; SPMs, specialized pro-resolving mediators.

**Table 4:** Comparative immune–damage management across species and longevity states.

Feature	Young Humans	Aged Humans	Centenarians	Bats	Long-Lived Mammals
Damage Tolerance	High (Baseline)	Low (Intolerant)	High (RNaseH2C-driven)	High but tolerated (S358 STING/PYHIN loss)	High but tolerated
Autophagy	High	Declines	Preserved	High basal	High
Proteostasis	High	Declines	Preserved (RNaseH2C)	Strong (HSP-mediated)	Enhanced
Basal Immune Readiness	Inducible	Dysregulated	Preserved	Constitutive	Preserved
Inflammasome	Low	Hyperactive	Restrained	Suppressed	Restrained
Inflammatory Resolution capacity	Efficient	Impaired	Preserved	Rapid	Efficient
Longevity	Normal	Reduced	Exceptional	Exceptional	Exceptional

**Table 5:** Nutritional strategies targeting the CLSV-6 framework: translation to human longevity.

CLSV-6 Domain	Representative Functional Foods & Human Dosage	Mechanistic Actions (Molecular Targets)	Representative Biomarkers (Human)	Translational Notes
Damage Tolerance	Berries: 1–2 servings/day; Green tea: 2–5 cups/day (300–800 mg catechins/EGCG); Cocoa flavanols: 200–600 mg/day; Extra-virgin olive oil: 20–40 g/day	NRF2 activation, DNA repair enhancement (PARP-1), STING suppression	8-OHdG, F2-isoprostanes, γ-H2AX	Benefits observed at dietary—rather than pharmacological—doses.
Autophagy–Mitophagy	Spermidine: 1–6 mg/day; Resveratrol: 150–500 mg/day; EGCG: 300–800 mg/day;	AMPK–ULK1 activation, mTOR inhibition, mitophagy induction	LC3-II/I ratio, Circulating Beclin-1, mtDNA copy number	Clears immunogenic metabolic waste before sensing occurs.
Proteostasis	Fermented foods: 1–3 servings/day; Quercetin: 500–1000 mg/day	Proteasome activation, Chaperone induction, UPR optimization	Circulating HSP70, Protein carbonyls	Preserves tissue function by preventing pro-inflammatory aggregation.
Basal Immune Readiness	Dietary fiber: 25–38 g/day; V-D: 1000–4000 IU/day; Zinc: 8–15 mg/day; Herbal adaptogens such as ginseng extracts: 0.5–3 g/day	SCFA production, IFN-tone maintenance, Barrier integrity support	IFN-stimulated gene expression, Salivary IgA, Vitamin D status	Supports “quiet vigilance” without systemic cytokine amplification.
Inflammasome Restraint	Curcumin: 500–1500 mg/day; Anthocyanin-rich berries: 1–2 servings/day or 300–600 mg anthocyanins/day; w-3 fatty acids (EPA + DHA): 1–3 g/day; ketogenic or fasting states	NLRP3 inhibition, Caspase-1 suppression, IL-1β reduction	hs-CRP (low-range), IL-1β/IL-10 ratio	Mimics the “bat-like” decoupling of sensing from inflammatory execution.
Inflammatory Resolution Capacity	Fatty fish: 2–4 servings/week; EPA + DHA 1–3 g/day; Extra-virgin olive oil: 20–40 g/day; Fermented foods: 1–3 servings/day	SPM synthesis (Resolvins/Protectins), Macrophage efferocytosis	SPM profiles, Serum IL-10, TGF-β	Promotes active inflammatory resolution rather than broad immune suppression.

Representative intake ranges reflect approximate exposures reported in human observational or intervention studies and may vary according to formulation, bioavailability, microbiome composition, and study population. NRF2, nuclear factor erythroid 2–related factor 2; PARP, poly(ADP-ribose) polymerase; AMPK, AMP-activated protein kinase; ULK1, Unc-51 like autophagy activating kinase 1; PINK1, PTEN-induced kinase 1; TFEB, transcription factor EB; UPR, unfolded protein response; IFN, interferon; STING, stimulator of interferon genes; NLRP3, NOD-like receptor family pyrin domain containing 3; SPMs, specialized pro-resolving mediators; SCFAs, short-chain fatty acids; CRP, C-reactive protein; IL-1β, interleukin-1 beta; IL-6, interleukin-6; mTOR, mechanistic target of rapamycin.

## Data Availability

Data can be provided upon request to the corresponding author.
